# Computational and Mathematical Methods in Medicine

**DOI:** 10.1155/2011/303089

**Published:** 2011-01-16

**Authors:** Pamela Jones, Sivabal Sivaloganathan

**Affiliations:** ^1^Institute of Molecular Medicine, Leeds University, Leeds LS9 7TF, UK; ^2^Department of Applied Mathematics, University of Waterloo, 200 University Avenue W., Waterloo, ON, Canada N2L 3G1

This issue marks a transition and a changing of the guard for Computational and Mathematical Methods in Medicine (CMMM). It is with some nostalgia that we look back on our long and illustrious association with Taylor and Francis; however, at the same time we look to the future with optimism and hope as Hindawi takes the helm and converts CMMM to the community-based, open access model that they have so successfully championed. The Hindawi Publishing Corporation is one of the fastest growing academic publishers worldwide with over 200 academic journals in their portfolio and a commitment to the highest levels of peer review and excellence. 

Reflecting on the genesis and evolution of CMMM, it is clear that Brian Sleeman, the founding Editor-in-Chief, showed great foresight in creating a journal that brought together the disparate disciplines of mathematics and medicine and that continues to play a major role in the development of mathematical medicine. He worked passionately to develop and promote the journal through some difficult times, with the insight and courage to bring together both biomedical/clinical scientists and mathematical scientists onto a single editorial board (a practice that has become more commonplace in subsequent journals in the field). The success that the journal has enjoyed thus far is a clear testament to his hard work, dedication, and vision. 

The journal has continued to provide a unique forum for the dissemination of interdisciplinary research resulting from collaborations between clinicians/experimentalists and theoreticians. CMMM has also continued to evolve rapidly, reflecting the increased focus on systems and interdisciplinary collaborative efforts across the breadth of biomedical, clinical, and translational research areas. The past year also saw the result of much hard work, with the inclusion of the journal in PubMed/Medline and the Science Citation Index Expanded. This was a great development for the journal since it not only has had an enormous impact on the general awareness and profile of the journal but has also resulted in increased submissions and downloads from the journal website over the past year. It has been exciting and rewarding to see the journal develop and evolve in this manner, and we look forward to increased success following this higher profile.

 The future looks extremely bright for the field of mathematical medicine as it emerges from its period of infancy and takes its place as a legitimate and central field of research and enquiry. Our sincere hope and wish is that CMMM continues from strength to strength and fulfills its role and promise as envisioned originally by its founding editor.

*Pamela Jones* *Sivabal Sivaloganathan*

